# The isothiocyanate erucin abrogates telomerase in hepatocellular carcinoma cells *in vitro* and in an orthotopic xenograft tumour model of HCC

**DOI:** 10.1111/jcmm.12412

**Published:** 2014-09-25

**Authors:** Corinna Herz, Anke Hertrampf, Stefan Zimmermann, Nadine Stetter, Meike Wagner, Claudia Kleinhans, Miriam Erlacher, Julia Schüler, Stefanie Platz, Sascha Rohn, Volker Mersch-Sundermann, Evelyn Lamy

**Affiliations:** a Institute for Environmental Health Sciences and Hospital Infection Control, Freiburg University Medical CenterFreiburg, Germany; b Faculty of Biology, University of FreiburgFreiburg, Germany; c Department of Hematology and Oncology, Freiburg University Medical CenterFreiburg, Germany; d Institute of Biological Chemistry and Nutrition, University of HohenheimStuttgart, Germany; e Department of Translational Oncology, University Medical Center MainzMainz, Germany; f Institute for Interfacial Engineering IGVT, University of StuttgartStuttgart, Germany; g Department of Paediatrics and Adolescent Medicine, Freiburg University Medical CenterFreiburg, Germany; h Department of Experimental Oncology, Oncotest GmbHFreiburg, Germany; i Hamburg School of Food Science, Institute of Food Chemistry, University of HamburgHamburg, Germany

**Keywords:** 4-methylthiobutyl isothiocyanate/erucin, sulforaphane, hTERT/telomerase suppression, hepatocellular carcinoma and chemoresistant subpopulations, adjuvant chemotherapy

## Abstract

In contrast to cancer cells, most normal human cells have no or low telomerase levels which makes it an attractive target for anti-cancer drugs. The small molecule sulforaphane from broccoli is known for its cancer therapeutic potential *in vitro* and *in vivo*. In animals and humans it was found to be quickly metabolized into 4-methylthiobutyl isothiocyanate (MTBITC, erucin) which we recently identified as strong selective apoptosis inducer in hepatocellular carcinoma (HCC) cells. Here, we investigated the relevance of telomerase abrogation for cytotoxic efficacy of MTBITC against HCC. The drug was effective against telomerase, independent from TP53 and MTBITC also blocked telomerase in chemoresistant subpopulations. By using an orthotopic human liver cancer xenograft model, we give first evidence that MTBITC at 50 mg/KG b.w./d significantly decreased telomerase activity *in vivo* without affecting enzyme activity of adjacent normal tissue. Upon drug exposure, telomerase decrease was consistent with a dose-dependent switch to anti-survival, cell arrest and apoptosis in our *in vitro* HCC models. Blocking telomerase by the specific inhibitor TMPyP4 further sensitized cancer cells to MTBITC-mediated cytotoxicity. Overexpression of hTERT, but not enzyme activity deficient DNhTERT, protected against apoptosis; neither DNA damage nor cytostasis induction by MTBITC was prevented by hTERT overexpression. These findings imply that telomerase enzyme activity does not protect against MTBITC-induced DNA damage but impacts signalling processes upstream of apoptosis execution level.

## Introduction

The hepatocellular carcinoma (HCC) is the fifth common cancer and the third leading cause of cancer related death worldwide [1]. Main factors for a bad survival rate are the non or poor responsiveness to chemo- and radiotherapy [2]. In this, highly treatment-resistant tumour-initiating cells that are capable of self-renewing and maintaining tumour growth, are thought to account for disease relapse after chemotherapy [3]. Another important determinant for cancer cell resistance is expression of anti-apoptotic factors such as telomerase [4–6]. Human telomerase reverse transcriptase (hTERT) represents the catalytic subunit of this enzyme. For executing its canonical function of telomere protection, hTERT needs association with an RNA template molecule (hTERC) and a number of telomerase associated proteins to form the active enzyme complex [7]. Besides, hTERT takes part in DNA repair [8,9] and protects cells from cell cycle arrest and/or apoptosis making cancer cells more resistant against chemotherapeutic agents or radiation therapy [5,10]. Current literature indicates that these non-canonical functions of hTERT either partly require enzyme activity but not telomere lengthening or neither of the two.

Sulforaphane (SFN), a natural small molecule isolated from plants of the family *Brassicaceae,* is known for its chemopreventive and -therapeutic actions both *in vitro* and *in vivo* [11–13] and based on promising preclinical findings, meanwhile clinical trials with SFN and broccoli sprouts are running for prevention or treatment of cancer (http://www.cancer.gov/clinicaltrials). It has also been clearly demonstrated that SFN is quickly metabolized to 4-methylthiobutyl isothiocyanate (MTBITC, erucin) *in vivo* [13] raising the question of biological activity of this metabolite. We recently demonstrated the preclinical efficacy of MTBITC against HCC and their chemoresistant subpopulations which was independent from TP53 [14]. Moreover, isothiocyanates (ITC) and in particular, SFN were shown as inhibitors of telomerase in different cancer cells [15–18]. Our own group found that mitogen-activated protein kinase pathway modulation by MTBITC is responsible for inhibition of hTERT gene expression in human HCC cells [19]. This discovery could have great implications for adjunctive liver cancer therapy by ITC in terms of cancer cell sensitization. Therefore, based on our previous findings, we now aimed to investigate whether enzyme activity loss upon ITC exposure is in fact an upstream mechanism or a downstream consequence of the apoptotic process in HCC-derived cells and their chemoresistant subpopulations. By using overexpression of hTERT and catalytically inactive hTERT mutants, the necessity of holenzyme activity for cell protection against MTBITC-induced DNA damage, cytostasis and consequently apoptosis was particularly addressed in this context. We finally wanted to provide first evidence for transferability of ITC-triggered telomerase activity inhibition observed *in vitro* to *in vivo* by using an orthotopic xenograft model of HCC.

## Materials and methods

DMSO (purity >99%), benzo(a)pyrene (purity 98%), propidium iodide (PI), phenylmethyl-sulfonylfluorid, etoposide, verapamil and valinomycin were acquired from Sigma-Aldrich (Steinheim, Germany). β-mercaptoethanol was from Merck (Darmstadt, Germany). CaCl_2_, glucose, EGTA and fomic acid (LC-MS-Grade) were acquired from Carl Roth (Karlsruhe, Germany) Dulbeccos Minimal Essential Medium (DMEM), foetal calf serum (FCS), trypsin 10× (25 mg/ml), trypsin-EDTA 10× (5 mg/ml respectively 2.2 mg/ml), PBS (without Ca and Mg), L-glutamine (200 mM) and Hanks balanced salt buffer (without Ca and Mg) were from PAA Laboratories GmbH (Coelbe, Germany). Hoechst 33342, DMEM, (low Glucose, without Phenol Red) and Penicillin/Streptomycin solution was purchased from Invitrogen (Darmstadt, Deutschland). Camptothecin from Tocris (Eching, Germany), Caspase 3/7 GLO reagent from Promega (Mannheim, Germany). Triton X-100 and Meso-5,10,15,20-Tetrakis(N-methyl-4-pyridyl) porphine, tetratosylate (TMPyP4) was from Merck (Mannheim, Germany). MTBITC was synthesized by the Institute of Organic Chemistry, University of Giessen, Germany as described elsewhere [20]. Acetonitrile (HPLC-grade) was from VWR (Darmstadt, Germany), C_18_ solid-phase extraction (cartridges, 1 ml, 100 mg) from Sigma-Aldrich (Taufkirchen, Germany) and Trifluoroacetic acid from Applichem (Darmstadt, Germany).

The following primary antibodies were used for immunoblotting: anti-Akt, anti-p-Akt (Ser 473, clone 587F11), anti-p-CHK2 (Thr68) and anti-p-CHK1 (Ser345), anti-p-H2A.X (Ser139,) were from Cell Signalling Technology (Boston, MA, USA); anti TP53 (clone BP53-12) and anti β-actin (clone AC-74) from Sigma-Aldrich; anti-hTERT (clone Y182) was from Biomol (Hamburg, Germany). The horseradish peroxidase-labelled secondary antibodies antimouse and anti-rabbit were purchased from Cell Signalling Technology (Danvers, MA, USA). Nuclease free water was from Qiagen (Hilden, Germany). MTBITC and benzo(a)pyrene were dissolved in sterile DMSO. TMPyP4 was dissolved in sterile double distilled water.

### HCC cell lines

HepG2 and Hep3B cell lines were obtained from the German Collection of Microorganisms and Cell Cultures (DSMZ, Braunschweig, Germany). Huh-7 cells were kindly provided by H. Blum (University Medical Center Freiburg, Germany). The cells were cultured in low glucose DMEM supplemented with 15% (HepG2) or 10% (Huh7, Hep3B) FCS and 1% penicillin-streptomycin in a 5% CO_2_ atmosphere at 37°C.

### Determination of drug effect

Drug effect was tested at cell passages from 4 to 10. For the experiments, cells were seeded and incubated for 48 hrs at 37°C, 5% CO_2_ atmosphere. After that, cells were exposed to MTBITC and subsequently processed for the assays.

### Single cell gel electrophoresis assay

Single cell gel electrophoresis assay, also known as comet assay, was carried out as described earlier [21]. The olive tail moment was calculated as indicator of DNA damage.

### Caspase 3/7 cleavage assay

Induction of apoptosis in cell lines was determined by using the Caspase3/7-Glo assay (Promega, Mannheim, Germany) according to the manufacturer's instructions.

### Phospho-ATM Activation

Ataxia-telangiectasia (ATM) activation was detected in HepG2 cells by using ATM Phospho Activation kit (Thermo Fisher Scientific, Rockville, MD, USA) according to the manufacturer's instructions. Cells were imaged by using a fluorescence microscope system 8100E from Keyence (Osaka, Japan) with an objective S PlanFluor ELWD 20×/0.45 (Nikon, Osaka, Japan).

### SubG1 DNA content and cell cycle distribution

For detection of cell cycle distribution, PI staining of DNA after fixation was used, as described elsewhere [22].

### Protein analysis by immunoblotting

Analysis of proteins by immunoblotting was performed as described before [19].

### RT-MLPA and qPCR

RT-MLPA was used to analyse mRNA expression of Bcl-2 family members. RNA from HepG2 cells was isolated as described before [14]. RT-MLPA (MRC Holland, kit RM002, R011-C1) was performed according to the manufacturer's instructions. Briefly, specific mRNAs were reversely transcribed into cDNA and bound by two oligonucleotides consequently ligated by a heat-stable ligase. Each probe gives rise to an amplification product of unique length separated by capillary sequencer (Genescan). Analysis was performed with GeneMapper (Applied Biosystems, Foster City, CA, USA). The sum of all peak data was set to 100% to normalize for fluctuations between different samples, and single peaks were calculated relative to 100%.

### Silencing of *TP53* by RNAi

HepG2 cells were transiently reverse transfected with siRNA oligomers for *TP53* (p53 ShortCut siRNA Mix, New England Biolabs, Frankfurt a. M., Germany; control siRNA, Santa Cruz Biotechnologies, Santa Cruz, CA, USA) at a final concentration of 25 nM and exposed to the test substance for 24 hrs as described elsewhere [14].

### Telomerase activity measurement by telomeric repeat amplification protocol-assay

Telomerase activity was detected by using the TeloTAGGG ELISA Kit from Roche (Mannheim, Germany) after the manufacturer's instructions with slight modifications described elsewhere [19]. Lysates from cell lines were prepared after the manufactures' instruction. To isolate proteins from xenograft tumours and normal mice liver, frozen tissue was cut on dry ice in small pieces, snap frozen in liquid nitrogen and crushed with a pestle. Afterwards, the tissue was resuspended in lyses buffer and homogenized with a needle for several times.

### Telomere length assessment by flow-FISH and Southern blot

Telomere length was determined by flow-FISH methodology by using the Telomere PNA kit/FITC from Dako (Glostrup, Denmark) according manufacture's introduction.

Additionally, telomere length was analysed by telomere restriction fragment (TRF) Southern blot by using the Telo*TAGGG* Telomere Length Assay Kit (Roche Diagnostics, Mannheim, Germany) according to the manufacturer's protocol. Mean telomere length was determined by densitometric analysis of autoradiographies by using the Telometric© 1.2 software (Biostatistics and Bioinformatics Facility, Fox Chase Cancer Center, Philadelphia, PA, USA).

### Retroviral Transfection of HepG2 cells

The pOS vector containing either the IRES-GFP or IRES-GFP-hTERT construct or a dominant-negative mutant of hTERT [23] were transfected into the Phoenix packaging cell line (kindly provided by G. Nolan, Stanford University, Stanford, CA, USA) to produce virus as previously described [24]. For transduction, HepG2 cells were grown for 48 hrs. Then fresh medium was added containing retroviral supernatant of Phoenix cells and 10 μg polyprene (Santa Cruz Biotechnologies, Santa Cruz, CA, USA). 48 hrs after transduction, cells were washed with PBS, trypsinized and GFP-positive cells were sorted by using a modular flow cytometer high-speed cell sorter (Cytomation, Fort Collins, CO, USA) and cultivated under standard conditions.

### Isolation and analysis of initiated tumour cells from HCC cells

Side population (SP) and non-SP cells were analysed and sorted from Huh7 cells as described before [14] by using Hoechst33342 staining and MoFlo sorter (DAKOCytomation). Expression of the enzyme aldehyde dehydrogenase (ALDH) was analysed by using the ALDEFLUOR Kit from Stemcell Technologies (Grenoble, France) according to manufacturer's protocol after 24 hrs exposure of Huh7 cells to MTBITC. Cell sorting was performed with a BD FACS Aria III Cell Sorter (San Jose, CA, USA).

### Orthotopic mouse xenograft model

All animal experiments were conducted according to the guidelines of the German Animal Welfare Act (Tierschutzgesetz) and under the permission numbers of the Regierungspräsidium Freiburg, Germany G-10/05 and 35-9185.64/1. Crl:NU-Foxn1mice (nude mice) were purchased from Charles River (Erkrath, Germany) and obtained in microisolators in barrier conditions. At 6–8 weeks of age, mice were anesthetized with isoflurane, Hep3B cells were resuspended in a volume of 100 μl PBS and injected into one liver lobe. Animals were randomized 21 days after tumour implantation and treatment was started by daily oral gavage with vehicle (sunflower oil) or MTBITC suspended in vehicle for another 21 days. For quantitative detection of tumour load, 3 days after implantation mice were injected intravenously with 0.02 mg anti-human-CD227-Antibody (AbD Serotec, Düsseldorf, Germany), that is labelled with fluorescence dye Xenolight CF750 (Caliper Life Sciences, Mainz, Germany). Mice were then anesthetized by isoflurane inhalation 20 hrs after the injection, and pictures were taken with a Bruker *in vivo* imaging system (Bruker Image Station *in vivo* FX pro). For optimal localization of the fluorescent region, animals were x-rayed and the two pictures merged. After the last treatment mice were sacrificed, livers removed and flash frozen in liquid nitrogen. To analyse MTBITC and its metabolites in blood plasma and mouse organs, 4 mice were treated by oral gavage with 50 mg/kg b.w. MTBITC. After 1 hr mice were scarified, blood plasma was isolated and liver as well as kidneys were removed and immediately flash frozen in liquid nitrogen.

### Analysis of MTBITC metabolite in mouse plasma and organs

For the analyses of MTBITC metabolite in mouse plasma, thawed samples were mixed for 1 min. and 100 μl used for analysis. After protein precipitation with 50 μl pre-cooled trifluoroacetic acid, the samples were centrifuged for 10 min. and 20,817 g at 4°C. For metabolite analyses in kidney and liver, the freeze-dried organs were homogenized and 10–50 mg of the powder extracted three times with 500 μl ACN-water (90:10, v/v, containing 0.1% formic acid). Subsequent to the individual sample preparation, the supernatants were used for solid-phase extraction as previously described [25]. The dried samples, deriving from plasma and organs were redissolved in 50 μl acetonitrile-water (85:15, v/v, containing 0.1% formic acid). Metabolite analysis by using LC-MS/MS was performed on an API2000 electrospray ionization triple quadrupole MS/MS system (Applied Biosystems) connected to an Agilent 1200 LC system (Agilent Technologies, Böblingen, Germany). Measurements were conducted in the positive-ion mode, and quantitative analysis was based on multiple reaction monitoring [26].

### Data analysis and statistics

Results were analysed by using GraphPad Prism 5 software (GraphPad Software Inc., LaJolla, CA, USA). Statistical significance was tested by Student's *t*-test and the variance between groups was tested by one-way anova. *P*-values of ≤0.05 and ≤0.01 were considered statistically significant.

## Results

### MTBITC blocks telomerase activity in HCC and chemoresistant subpopulations

**Fig. 1:**
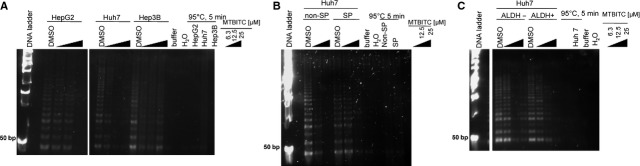
MTBITC decreases telomerase activity in HCC cells and their chemoresistant subpopulations. Cells were exposed to MTBITC or solvent (0.1% DMSO) for 24 hrs. Whole protein lysates were used for TRAP-assay and the product was separated on a polyacrylamid gel and stained with SYBR gold. Lyses buffer, nuclease free water (H_2_O) and heat treated samples of each cell population were included as TRAP negative controls. For molecular weight determination, a 50 bp DNA ladder was used. Concentration dependent telomerase activity inhibition was evident in (**A**) HCC cell lines, (**B**) non-SP and SP cells from Huh7, (**C**) ALDH-negative and -positive cells from Huh7. The pictures are representatives from independent experiments. SP, side population; HCC, hepatocellular carcinoma; TRAP, telomeric repeat amplification protocol.

At first we studied the concentration dependent effect of MTBITC on telomerase activity of wt-TP53 (HepG2), mut-TP53 (Huh7) and null-TP53 (Hep3B) cells after 24 hrs. As shown in Figure 1A, 24 hrs-exposure of cells to MTBITC reduced telomerase activity at concentrations ≥6.3 μM. This observation was independent from the cell line under study. Abrogation of telomerase activity was also observed in chemoresistant SP and ALDH-positive cells (Fig. 1B and C), but non-SP cells from Huh7 were more sensitive than SP-cells (Fig. 1B). For the marker ALDH, no such differences were seen, neither in Huh7 (Fig. 1C) nor HepG2 (data not shown).

### Telomerase abrogation by MTBITC is independent of TP53

**Fig. 2:**
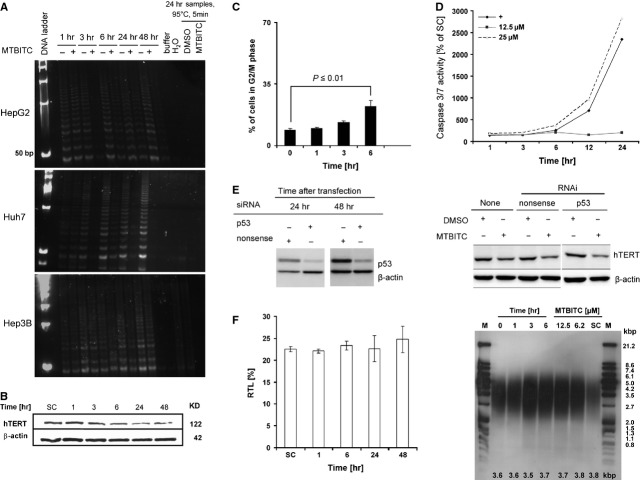
MTBITC abrogates telomerase in HCC cells independent from TP53. (**A**) Time-dependent inhibition of telomerase activity in HCC cells after exposure to 25 μM MTBITC (+) or 0.1% DMSO (−) for 1–48 hrs. HCC protein lysates were used for TRAP-assay. Lyses buffer and nuclease free water (H_2_O) as well as heat treated samples were included as TRAP negative controls. (**B**) Representative immunoblot of time dependent hTERT protein suppression after exposure of HepG2 cells to 25 μM MTBITC for 1–48 hrs. β-actin was used as loading control. (**C**) Time course of G2/M phase arresting HepG2 cells after treatment with 25 μM MTBITC as analysed by PI staining of DNA and flow cytometry. Bars are mean + SD (*n* = 3). (**D**) Time course of MTBITC-mediated apoptosis induction in HepG2 cells, as determined by the Caspase-Glo® 3/7 assay. The results were expressed as percentage of control cells, treated for the indicated time points with 0.1% DMSO. +, positive control = 10 μM camptothecin (*n* = 3). (**E**) Silencing of *TP53* in HepG2 cells by using RNAi. HepG2 cells were reverse transfected with *TP53*-RNAi or nonsense-RNAi for the indicated time points. Then, cell lysates were screened for down regulation of TP53 by immunoblotting by using an antibody against TP53 (left pannel). Impact of transient *TP53* knockdown on MTBITC-induced hTERT suppression. Cells were either untransfected, reverse transfected with nonsense-RNAi or *TP53*-RNAi for 24 hrs and subsequently exposed to 0.1% DMSO or 25 μM MTBITC for another 24 hrs. Cell lysates were then prepared for immunoblotting by using an antibody against hTERT. β-actin was used as loading control (right pannel). (**F**) MTBITC did not acutely affect telomere length of HepG2 cells. Relative telomere length (RTL) of cells treated with 25 μM MTBITC for the indicated time points. bars are mean ± SD, *n* = 3 (left pannel). TRF Southern blot analysis of HepG2 cells, exposed to 25 μM MTBITC for 1 to 6 hrs or to different concentrations for 6 hrs. SC: solvent control = 0.1% DMSO. M: Molecular weight marker. Numbers in the panel indicate mean telomere length in kbp (right panel).

In the next step, the time course of telomerase abrogation upon drug-exposure was assessed in HCC cells (Fig. 2A). Within 3 hrs, MTBITC-treatment could potently abolish telomerase activity in all three cell lines. The effect was also detectable on hTERT protein level, representatively analysed in wt-TP53 (HepG2) cells (Fig. 2B). To put these observations in the time context of growth arrest and apoptosis, we further investigated the over-time G2/M arrest (Fig. 2C) and caspase 3/7 activation (Fig. 2D) in wt-TP53 cells after drug exposure. G2/M halt was evident at 3 hrs, caspase activity increase not before 6 hrs after MTBITC exposure. Although activated by MTBITC, it was shown previously by us that cytotoxicity of MTBITC was TP53 independent [14]. TP53 is an important negative transcriptional regulator of telomerase and we therefore next investigated its relevance for telomerase suppression by MTBITC in wt-TP53 cells. Efficient p53 knockdown by RNAi is shown in Figure 2E (left). As evident from Figure 2E (right), *TP53*-RNAi could not protect the cells from hTERT degradation, triggered by MTBITC.

Acute loss of telomeric repeat sequences because of the uncapping of telomeres can precede apoptosis induction [27]. In most studies so far investigating the anti-apoptotic role of telomerase, it remained unclear, whether cell death induction occurred in a process involving telomere disruption. We analysed the telomere length in HepG2 cells after MTBITC treatment by using flow-FISH analysis. No significant change in mean telomere length could be observed by drug treatment (Fig. 2F, left panel). For confirmation of these results, we used specific TRF analysis. Mean telomere length of cells was unaffected by cytotoxic concentrations of MTBITC (Fig. 2F, right panel).

### Telomerase abrogation sensitizes cells to apoptosis by MTBITC

**Fig. 3:**
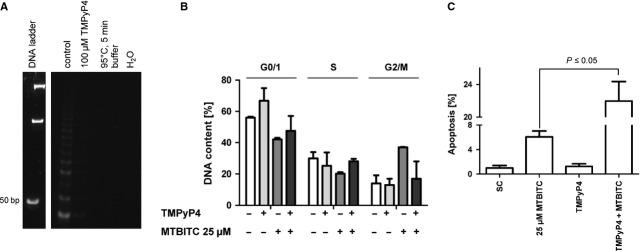
Telomerase enzyme loss sensitizes cells to MTBITC-triggered apoptosis. HepG2 cells were pre-treated with 100 μM TMPyP4 for 48 hrs and subsequently exposed to MTBITC for another 24 hrs. (**A**) Telomerase inhibition by TMPyP4 was verified by using the TRAP-assay. Lyses buffer, nuclease free water (H_2_O) and heat treated samples were included as TRAP negative controls. control = cells treated with solvent (1% H_2_O) for 48 hrs. (**B**) cell cycle arrest and (**C**) apoptosis induction by MTBITC as determined by PI staining of DNA and flow cytometric analysis. SC, solvent control = 1% H_2_O and 0.1% DMSO. Cells exposed only to 25 μM MTBITC for 24 hrs were included as reference, bars are mean ± SD (*n* = 2).

We next investigated whether telomerase abrogation by the drug substantially contributed to cancer cell sensitization for apoptosis. To address this we analysed the DNA content of HCC cells in the presence of the telomerase inhibitor TMPyP4. Enzyme activity was completely abrogated in HepG2 cells, treated with 100 μM TMPyP4 after 48 hrs (Fig. 3A). Consequently, inhibitor pre-treatment led to abolishment of MTBITC-mediated G2/M halt (Fig. 3B) and strong enhancement of apoptosis induction (Fig. 3C).

### Overexpression of hTERT but not DNhTERT decreases MTBITC-induced apoptosis

**Fig. 4:**
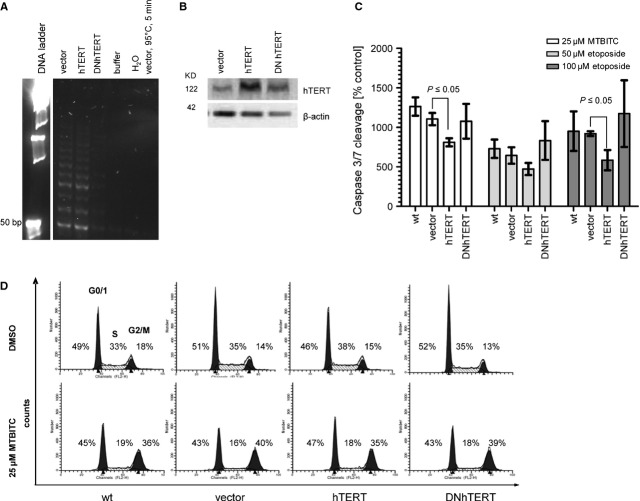
hTERT but not DNhTERT overexpression blocks MTBITC mediated apoptosis. HepG2 cells were stably transfected by using a lentiviral system with pOS-IRES-GFP (vetor), pOS-IRES-GFP-hTERT (hTERT) or pOS-IRES-GFP-DNhTERT (DNhTERT). GFP positve cells were sorted and seeded into cell culture dishes. Verification of efficient transfection was done by quantification of (**A**) telomerase activity; protein lysates were used for TRAP-assay. Lyses buffer and nuclease free water (H_2_O) as well as heat treated samples were included as TRAP negative controls. (**B**) hTERT protein level by immunoblot by using a monoclonal antibody against hTERT; beta-actin was used as loading control. (**C**) MTBITC-mediated apoptosis in HepG2 cells were determined by the Caspase-Glo® 3/7 assay. Results were expressed as percentage of control, treated for the indicated time-points with 0.1% DMSO. 50 and 100 μM etoposide for 24 hrs was used as reference (*n* = 5). (**D**) DNA content analysis by propidium iodide staining and flow cytometry of HepG2 cells after treatment with 25 μM MTBITC. Representative histograms are shown (*n* = 3).

To clarify if hTERT acts anti-apoptotic against drug-induced cytostasis and cytotoxicity, we generated HepG2 clones which either overexpress hTERT or dominant-negative hTERT (DNhTERT) devoid of telomerase enzyme activity. Successful cloning was verified by enzyme activity analysis (Fig. 4A), immunoblotting against hTERT (Fig. 4B) and qRT-PCR (data not shown). As depicted in Figure 4C, overexpression of hTERT led to a statistically significant (*P* < 0.05) apoptosis abrogation in MTBITC-treated cells as compared to control vector. This effect was not evident in DNhTERT clones. Besides MTBITC, we used the topoisomerase II inhibitor etoposide as reference. With this, the same effect was observed (Fig. 4C). Interestingly, hTERT or DNhTERT overexpression did not impact MTBITC-triggered cell halt (Fig. 4D). To further investigate the mechanism of decreased apoptosis in hTERT overexpressed cells, we analysed the BCL pathway by MPLA; but no significant change of genes in this pathway compared to vector cells was evident (data not shown).

### MTBITC treatment causes activation of the ATM/CHK2 DNA damage pathway

**Fig. 5:**
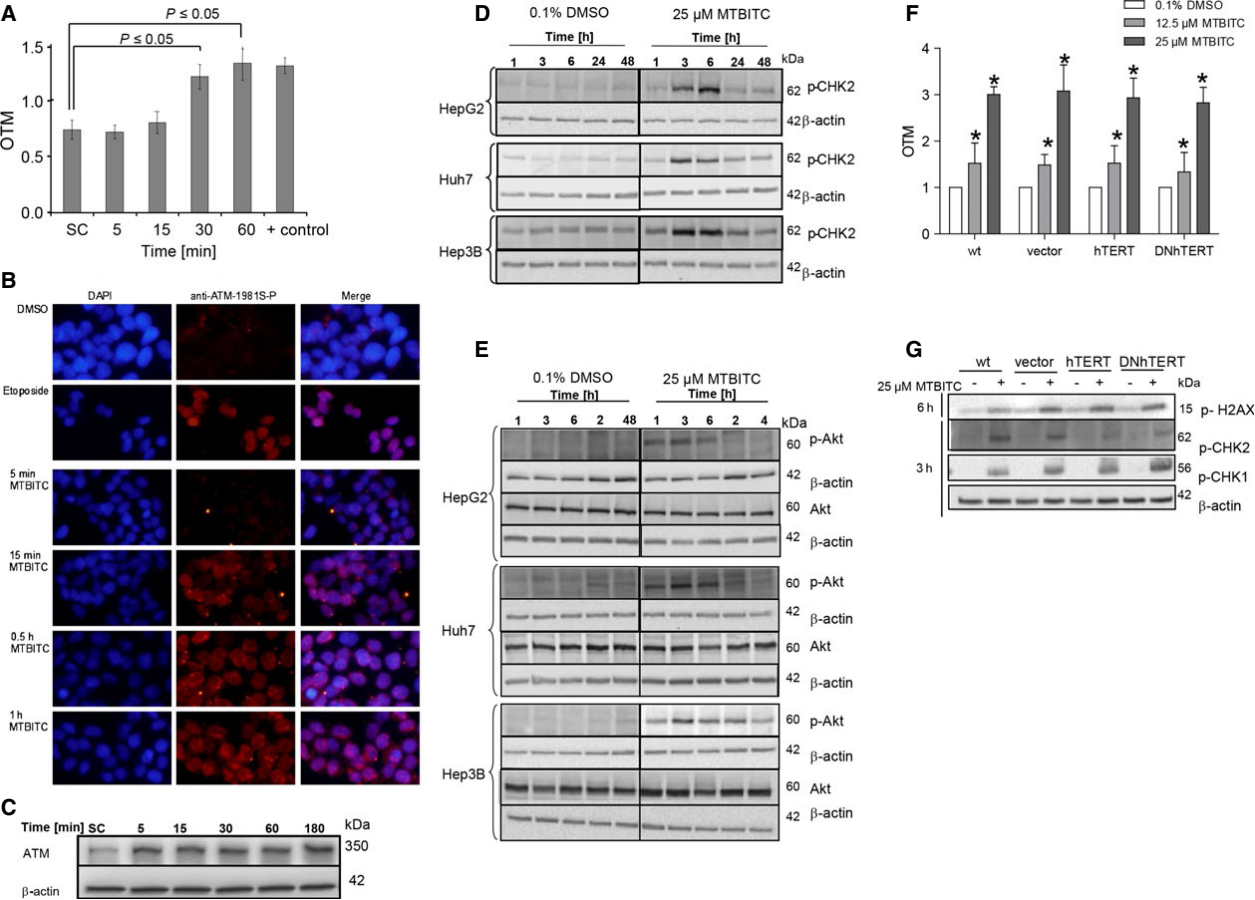
DNA damage pathway activation by MTBITC. (**A**) Time course of DNA damage induced by MTBITC in HepG2 cells as assessed by the comet assay. SC, solvent control = 0.1% DMSO; +, positive control = 100 μM benzo(a)pyrene (*n* = 3). OTM, olive tail momment (**B**) MTBITC activates ATM in HepG2 cells. Cells were treated with vehicle (0.1% DMSO) or 25 μM MTBITC for 5–60 min. Phospo-ATM was stained by using an antibody against ATM Ser1981. Cells were evaluated under a fluorescence microscope from Keyence, Germany. Treatment of cells with 100 μM etoposide for 1 hr was used as positive control. (**C**) Representative immunoblot of ATM protein accumulation after exposure of HepG2 cells to 25 μM MTBITC. (**D**) Representative immunoblot of Akt activation and total Akt protein level in HCC cells after 25 μM MTBITC or 0.1% DMSO treatment. (**E**) Representative immunoblot of CHK2 activation in HCC cells after treatment with 25 μM MTBITC or 0.1% DMSO for 1–48 hrs. β-actin was used as loading control. (**F**) DNA damage induced by MTBITC as assed by comet assay. Bars are mean ± SEM, *n* = 5. (**G**) Representative immunoblot of gamma-H2AX, CHK2 and CHK1 activation in HCC cells after treatment with 25 μM MTBITC or 0.1% DMSO for 1–48 hrs. HepG2 cells were stable transduced by using lentiviral system with pOS IRES GFP (vetor), pOS IRES GFP_hTERT (hTERT) and pOS IRES GFP_DN_hTERT (DN hTERT). wt, HepG2 cells. β-actin was used as loading control.

Induction of DNA strand breaks in response to MTBITC exposure was monitored by the comet assay (Fig. 5A). Within 30 min. of MTBITC treatment, a clear increase in DNA damage was visible. Further proof was given by the early detection of p^Ser1981^ATM (Fig. 5B) concurring with an accumulation of total ATM protein level (Fig. 5C) and p^Thr68^CHK2 (Fig. 5D) - both phosphorylations are known markers of double strand breaks. Activated CHK2 is then capable of regulating cell cycle progression and/or apoptosis [28]. CHK2, activated at 3 hrs (Fig. 5D), was evident in all three HCC cell lines. Because ATM mediates activation of Akt in response to cellular stress, we additionally investigated its response to MTBITC. Akt phosphorylation at Ser473 was increased immediately in treated HCC cells (Fig. 5E) which can be considered as compensatory protective mechanism by the cell to escape death. However, this activation was turned off about 6 hrs after treatment in wt-TP53 cells but only at 24 hrs in del-TP53 cells.

### hTERT overexpression did not impact MTBITC-induced DNA damage

Next, we studied the effect of hTERT overexpression on MTBITC-induced DNA damage. No difference in MTBITC-induced DNA damage was detected in hTERT overexpressed or DNhTERT HepG2 cells as compared to vector control after 24 hrs (Fig. 5F). As another parameter for DNA damage induction, phosporylation at Ser139 of H2A.X was used after 6 hrs treatment of MTBITC. Again, no difference was seen between the cell clones (Fig. 5G). Compared to vector control, activation of CHK2 was blocked in both, hTERT und DNhTERT overexpressed cell lines upon MTBITC-treatment. Elevated levels of p^Thr68^CHK1 were only detected in DNhTERT clones upon MTBITC exposure (Fig. 5G).

### *In vivo* inhibition of telomerase by MTBITC

**Fig. 6:**
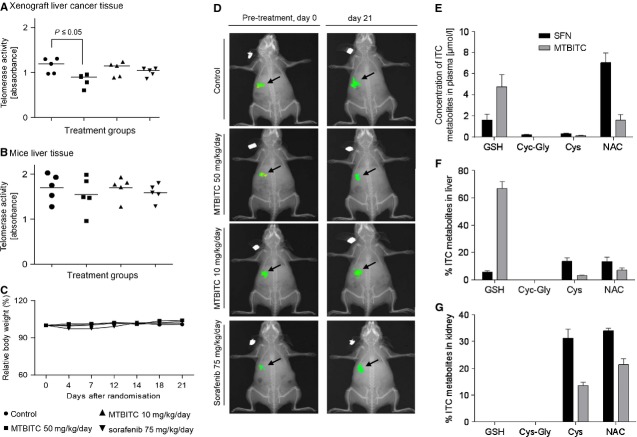
MTBITC decreases telomerase actvity levels in Hep3B tumours grown *in vivo*. Nude mice were inoculated with 5–6 × 10^6^ Hep3B cells, randomized into groups and treated for 3 weeks by daily gavage starting on day 21 with vehicle, MTBITC (50 mg/kg/day) or (10 mg/kg/day), sorafenib (75 mg/kg/day). Measurement of telomerase activity in the xenograft tumour tissue (**A**) and normal mice liver (**B**) tissue by using PCR based TRAP-ELISA assay, *n* = 5 each group. (**C**) Time course of relative mean bodyweight. The bodyweight was set at day 0 (start of gavage) to 100% for each group and plotted for data gathered at days 4–21, *n* = 6 each group. (**D**) *In vivo* imaging pictures, for representative animals from each group that received Hep3B cells at the indicated treatment days are displayed. These were taken with a Kodak Image Station merged with x-ray pictures of the animals. For quantification of MTBITC metabolites in plasma, mice liver and kidney, nude mice were given MTBITC at 50 mg/KG b.w. by oral gavage. After 1 hr, samples were taken and flash frozen in liquid nitrogen. MTBITC metabolites were analysed by LC-MS/MS in (**E**) blood plasma (**F**) liver and (**G**) kidney. Bars are mean ± SD, *n* = 4. SFN, sulforaphane; GSH, glutathione; Cys, cysteine; CysGly, cysteinyl glycine; NAC, N-acetylcysteine conjungates.

Based on our results derived *in vitro* we then determined whether orally applied MTBITC could also inhibit telomerase activity in HCC *in vivo*. As reference therapeutic, sorafenib (Nexavar™), a multi-kinase inhibitor directed against a broad range of protein kinases was used. By today, this is the only chemotherapeutic approved for the treatment of advanced HCC. At a dose of 50 mg/KG b.w./d MTBITC a significant (*P* < 0.05) decrease in telomerase activity was evident in xenograft tissue (Fig. 6A) but not in adjacent normal mice liver tissues (Fig. 6B). No difference was seen at the lower dose of MTBITC or the sorafenib group in both tissues. Bodyweights of the vehicle control, MTBITC or sorafenib-treated mice did not differ throughout the experimental period indicating good tolerability (Fig. 6C). At the end of day 21, a weak anti-tumour activity in terms of reduced tumour size of orthotopically growing Hep3B cells was found in animals treated with the high dose of MTBITC (tumour size MTBITC 50 mg/kg/day: 6.2 × 10^6^ ± 2.6 × 10^6^ pixel absolute net intensity, vehicle 7.1 × 10^6^ ± 3 × 10^6^ pixel absolute net intensity, Fig. 6D). The tumour load in the sorafenib-treated group was slightly enhanced at this time-point. However, none of these observations were statistically significant.

### MTBITC is quickly absorbed from the gastrointestinal tract, oxidized to SFN and metabolized by the mercapturic acid pathway

To study uptake and metabolism of MTBITC in the mouse model, we quantified MTBITC and SFN metabolites in plasma, kidney and liver 1 hr after oral application of MTBITC by using LCMS/MS. In plasma and mouse tissue, MTBITC and SFN metabolites were readily detectable (Fig. 6E–G). Intense conversion of MTBITC to SFN was evident (Fig. 6E). The most abundant ITC metabolite in plasma was SFN-NAC and MTBITC-GSH, both in the micro molar range. In liver tissue, MTBITC-GSH (Fig. 6F) and in the kidney, SFN-Cys and SFN-NAC (Fig. 6G) were the most abundant metabolites.

## Discussion

The role of telomerase in cell cycle regulation and its importance in the vulnerability to drug-induced apoptosis in cancer cells is not well understood. However, clear evidence is given that hTERT expression impacts cells cycle progression, as well as resistance to apoptosis induction (for review see Ref. [4]). Lately, it was discovered that the naturally occurring SFN also triggers telomerase down-regulation upon growth suppression of different human cancer cells [15–18,29]. So far, the relevance of this step for cancer cell cytotoxicity in general and HCC in particular was unknown. Our present findings demonstrated a gradual, concentration, as well as time-dependent loss of telomerase enzyme activity, triggered by MTBITC. Our data furthermore indicate that telomerase inhibition by MTBITC is also a relevant mechanism in chemoresistant subpopulations of HCC. This finding correlates well with recently published data of our group which showed selective cytotoxic action of MTBITC not only against the bulk tumour cells but also subpopulations positive for ALDH or Hoechst 33342 dye exclusion as marker for chemoresistance [14]. Moreover, loss of telomerase upon MTBITC treatment was demonstrated in our study to be independent from TP53. This is consistent with our earlier findings, which clearly showed TP53 independence of MTBITC *in vitro* therapeutic efficacy against HCC [14]. This tumour suppressor is one of the most important gate keeper and downstream effectors for G2/M cell cycle arrest or apoptosis [30]. Overexpressed wild-type TP53 has also been verified to transcriptional suppress hTERT activation [31,32] by binding of stabilized TP53 with the assistance of SP1 to the hTERT promotor [33]. However, in the case of HCC, no correlation was found between telomerase expression status in tumour tissue from patients and TP53 overexpression [34]. This and other data, suggesting that the regulatory function of TP53 on hTERT is rather cell type specific [35] are in support of our own study. Altogether our data suggest that telomerase inhibition is a common mechanism in MTBITC-triggered cytotoxicity in HCC and that the drug might even effectively target HCC cells that contribute to relapse and metastasis.

Isothiocyanates, including SFN and MTBITC have been demonstrated as DNA breaking agents in numerous cancer cell models [36]. In an earlier study on HepG2 cells, we found that up to a threshold dose, this DNA damage was subjected to intense repair, completely restoring DNA integrity within short time; but with increasing concentration, damage outweighed this repair capacity [22]. Our present results now show that initial levels of such DNA strand breaks trigger ATM and Akt pro-survival signalling. At a later stage, Akt is turned off and telomerase enzyme activity loss consistent with a dose-dependent switch to anti-survival, G2/M arrest and apoptosis. Besides other factors, the DNA damage response pathway has recently been found to be regulated by hTERT. So, hTERT overexpression strongly enhanced cellular repair, its suppression abrogated the response to DNA damage subjecting cells to death [37]. Our data indicate that hTERT did not protect HCC cells from DNA damage in terms of increased DNA repair, but prevented cells from caspase 3 dependent apoptosis, as observed before [5,38]. Moreover, telomerase enzyme activity seems to be indeed relevant for cell protection against drug-induced apoptosis. This is based on the observation that – in contrast to enzyme deficient cell clones – caspase activation was strongly diminished in hTERT overexpressed HepG2 cells. Enzyme activity abrogation by pre-treatment with a chemical inhibitor even further sensitized cells to MTBITC-triggered cell death. Interestingly, G2/M cell block was not impacted by hTERT overexpression suggesting that cytostasis induction by MTBITC is independent from telomerase level of tumour cells.

In the present study, we could provide first evidence for transferability of ITC-triggered telomerase activity inhibition observed *in vitro* to *in vivo* as this was found in Hep3B tumours orthotopically grown in livers of mice; no such effect could be detected in adjacent mouse liver tissue which might indicate a tumour cell selective action. But this observation needs further investigation because of a relatively small sample size in our study. The presence of both MTBITC and SFN metabolites in plasma and key tissues that are directly related to ITC metabolism confirms that MTBITC is quickly oxidized to SFN *in vivo*. Because therapeutic efficacy of MTBITC was better than SFN in a heterotrophic bladder tumour mouse model [39], the usage of MTBITC instead of SFN could be of therapeutic advantage but we must continue to explore this.

In conclusion, this pre-clinical study demonstrates that telomerase inhibition by MTBITC is a sensitizing step in cytotoxicity to HCC-derived cells. Telomerase abrogation thereby occurred at concentrations, which were clearly not toxic to healthy human hepatocytes [14]. This is the first step towards closing the knowledge gab which existed up to now on the chemotherapeutic potential of ITC against HCC with respect to telomerase inhibition. Lately, evidence was given that ITC act as chemotherapeutic sensitizers (see for review Ref. [40]); therapeutic trials by using combinations of botanicals such as curcumin [41] or chemotherapeutic agents, *e.g*. docetaxel [42] with ITC have shown that synergy between the agents can lead to lower doses, improved efficacy and fewer or less severe toxicities. On the basis of our present *in vivo* results, we therefore suggest that this should be further examined, both in terms of evaluating the optimal dosing of MTBITC against liver cancer and the possibility of its reinforcement of other chemotherapeutic agents.
